# Allogeneic human umbilical cord-derived mesenchymal stem cells for severe bronchopulmonary dysplasia in children: study protocol for a randomized controlled trial (MSC-BPD trial)

**DOI:** 10.1186/s13063-019-3935-x

**Published:** 2020-01-31

**Authors:** Xian Wu, Yunqiu Xia, Ou Zhou, Yan Song, Xianhong Zhang, Daiyin Tian, Qubei Li, Chang Shu, Enmei Liu, Xiaoping Yuan, Ling He, Chengjun Liu, Jing Li, Xiaohua Liang, Ke Yang, Zhou Fu, Lin Zou, Lei Bao, Jihong Dai

**Affiliations:** 1 Pediatric Research Institute, Children’s Hospital of Chongqing Medical University, Ministry of Education Key Laboratory of Child Development and Disorders, No 136, 2nd Zhongshan Rd, Yuzhong District, Chongqing, 400014 China; 2 Chongqing Key Laboratory of Pediatrics, Chongqing, China; 3 China International Science and Technology Cooperation Base of Child Development and Critical Disorders, Chongqing, 400014 China; 4 Department of Neonatology, Children’s Hospital of Chongqing Medical University, Chongqing, 400014 China; 5 Department of Respiratory Medicine, Children’s Hospital of Chongqing Medical University, Chongqing, 400014 China; 6 Department of Radiology, Children’s Hospital of Chongqing Medical University, Chongqing, 400014 China; 7 Department of Critical Care Medicine, Children’s Hospital of Chongqing Medical University, Chongqing, 400014 China; 8 Statistical Laboratory, Children’s Hospital of Chongqing Medical University, Chongqing, 400014 China; 9 Chongqing Engineering Research Center of Stem Cell Therapy, Chongqing, 400014 China; 10 Center for Clinical Molecular Medicine, Children’s Hospital of Chongqing Medical University, Chongqing, 400014 China

**Keywords:** Bronchopulmonary dysplasia, Human umbilical cord-derived mesenchymal stem cells, Clinical trial, Protocol

## Abstract

**Background:**

Bronchopulmonary dysplasia (BPD) is a complex lung pathological lesion secondary to multiple factors and one of the most common chronic lung diseases. It has a poor prognosis, especially in preterm infants. However, effective therapies for this disease are lacking. Stem-cell therapy is a promising way to improve lung injury and abnormal alveolarization, and the human umbilical cord (hUC) is a good source of mesenchymal stem cells (MSCs), which have demonstrated efficacy in other diseases. We hypothesized that intravenously administered allogeneic hUC-MSCs are safe and effective for severe BPD.

**Methods:**

The MSC-BPD trial is a randomized, single-center, open-label, dose-escalation, phase-II trial designed to investigate the safety and efficacy of hUC-MSCs in children with severe BPD. In this study, 72 patients will be enrolled and randomly divided into two intervention groups and one control group. Patients in the intervention groups will receive a low dose of hUC-MSCs (*n* = 24; 2.5 million cells/kg) or a high dose of hUC-MSCs (*n* = 24; 5 million cells/kg) in combination with traditional supportive treatments for BPD. The patients in the control group (*n* = 24) will be treated with traditional supportive treatments alone without hUC-MSCs. The primary outcome measures will be cumulative duration of oxygen therapy. Follow-up assessments will be performed at 1, 3, 6, 12, and 24 months post intervention, and the key outcome during follow-up will be changes on chest radiography. Statistical analyses will evaluate the efficacy of the hUC-MSC treatment.

**Discussion:**

This will be the first randomized controlled trial to evaluate the safety and efficacy of intravenously administered hUC-MSCs in children with severe BPD. Its results should provide a new evidence-based therapy for severe BPD.

**Trial registration:**

ClinicalTrials.gov, ID: NCT03601416. Registered on 26 July 2018.

## Background

Bronchopulmonary dysplasia (BPD) is a common chronic lung disease whose incidence is increasing annually, especially in this time of the two-child policy in China [1, 2]. Patients with BPD usually have ventilator or oxygen dependence during the early stage of the disease [3]. Most patients can gradually withdraw from the ventilator or stop oxygen treatment at different times depending on disease severity, but abnormalities in pulmonary structure and lung function may last until late childhood and even adulthood, especially in those who are diagnosed as severe BPD according to the diagnostic criteria of the National Institute of Child Health and Human Development (NICHD) [4, 5]. The mortality of overall BPD patients is about 15% [6]; nevertheless, the mortality rate of severe BPD reaches 41%, creating an enormous threat to the health of these children [4, 7]. More than 50% of survivors with BPD experience hospital readmission for repeated lower respiratory infections in the first year or two, which causes serious economic and labor burdens on families [4, 8]. Although surfactant treatment [9], prenatal steroid usage [1, 10], ventilator strategies [11], and improved nutrition [12] are used in BPD patients, effective therapies are lacking [13]. Therefore, identifying novel effective therapies for severe BPD in children is urgent and significant.

In recent years, the rapid development of stem-cell technology and regenerative medicine has identified stem cells as potential treatments for various refractory diseases, which are difficult to treat by traditional medical methods, such as degenerative diseases, cancer, and tissue damage [14–16]. Mesenchymal stem cells (MSCs) are a class of adult stem cells derived from the mesoderm with characteristics of non-tumorigenicity, low immunogenicity, and powerful paracrine function, and can be isolated from several sources, including bone marrow, human umbilical cord (UC), adipose tissue, amniotic fluid, and other tissues [17, 18]. Among them, human umbilical cord (hUC)-derived mesenchymal stem cells (hUC-MSCs) are useful in clinical application because they are easy to obtain, more proliferative, and have more powerful paracrine function than any other sources; they are effective at relieving lung inflammation, fibrosis, angiogenesis, and apoptosis [19–23]. Interestingly, the therapeutic potential of hUC-MSCs in several animal pulmonary disease models, including BPD, acute lung injury, and idiopathic pulmonary fibrosis, were confirmed [21, 24, 25]. Approximately 75% of hUC-MSCs reportedly accumulate in the microvessels of the lungs [26]. Hence, the therapeutic potential of hUC-MSCs for severe BPD in children requires investigation.

A multicenter, dose-escalation, phase-I clinical trial (NCT01775774) investigated the safety and efficacy of bone-marrow-derived MSCs for moderate to severe acute respiratory distress syndrome (ARDS) in adults without any adverse events (AEs) related to infusion reported, which had potential efficacy [27]. The study reported that hUC-MSC treatment was safe in patients with moderate to severe chronic obstructive pulmonary disease (COPD) (NCT00683722) [28]. Another phase-I trial reported treating nine BPD patients in Korea with hUC-blood-derived mesenchymal stem cells (hUCB-MSCs) (NCT01297205) [29]. The follow-up data from this study showed the safety of hUCB-MSC administration and the ability to reduce the level of profibrotic factors in tracheal aspirates [30]. Despite these data indicating the safety of MSC infusions for patients with pulmonary diseases, the limited sample size and lack of appropriate controls in those trials were insufficient to show the efficacy of MSC treatment. A large sample size of phase-II trials with matched controls is required to further investigate hUC-MSC safety and efficacy. Based on the previous promising findings, we designed an MSC-BPD trial to evaluate the safety and efficacy of the intravenous infusion of hUC-MSCs in children with severe BPD.

## Methods

### Study objectives

The goal of this clinical trial is to test the safety and efficacy of hUC-MSCs in children with severe BPD. There are three specific objectives:

1. Evaluate the long-term safety and efficacy of intravenously administered hUC-MSCs in children with severe BPD
2. Test the hypothesis that the administration of hUC-MSCs can reduce the duration of mechanical ventilation and oxygen and improve impairment of the pulmonary structure in children with severe BPD; and
3. Explore the potential therapeutic mechanism of hUC-MSCs for severe BPD

### Study design and setting

**Fig. 1:**
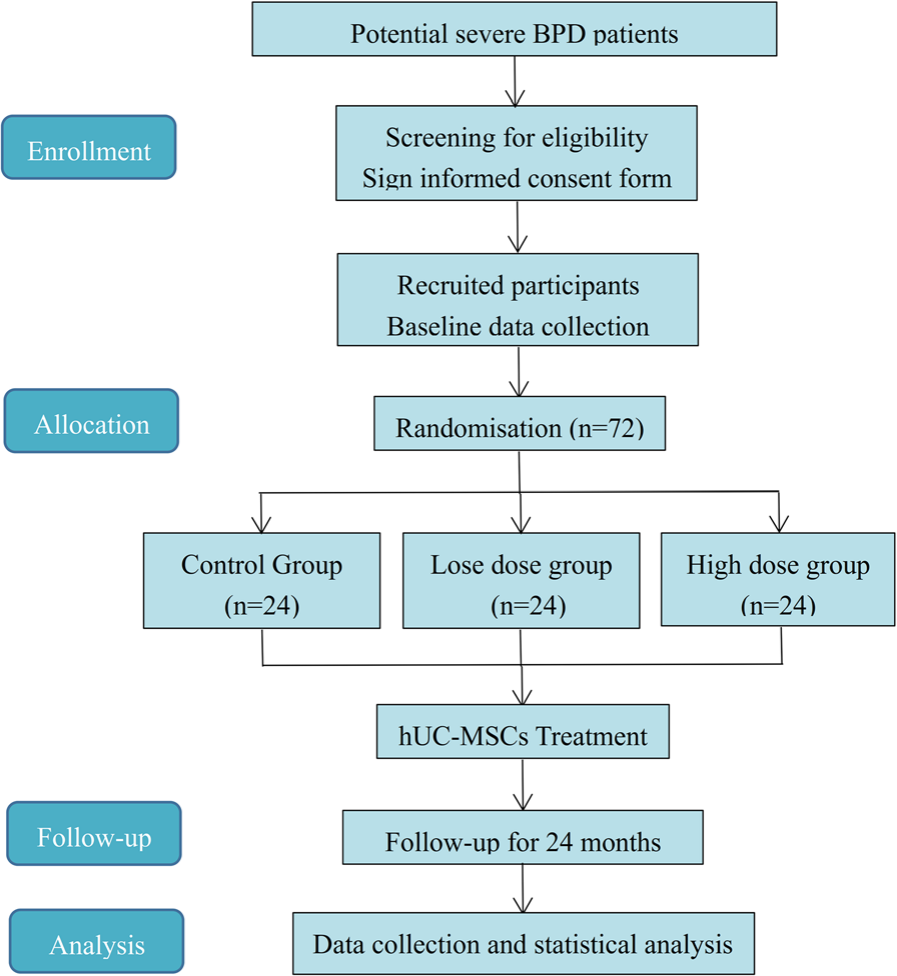
Study flow diagram

The MSC-BPD trial (registered at www.clinicaltrials.gov (no. NCT03601416) is a randomized, single-center, dose-escalation, phase-II trial aiming to evaluate the safety and efficacy of hUC-MSCs in a total of 72 children with severe BPD. A study flow chart of the trial is shown in Fig. 1. The study protocol will be reported based on Standard Protocol Items: Recommendations for Interventional Trials (SPIRIT) guidelines (Additional file 1).

This trial will be conducted at the Children’s Hospital of Chongqing Medical University (CHCMU) in Chongqing, China.

### Sample size and calculation

The sample size of this phase-II randomized controlled trial was calculated by power analysis using the Power and Sample Size online calculator (http://powerandsamplesize.com/). The trial is designed to investigate the hypotheses of two interventions compared to control, but it is not powered to test differences between the two intervention groups. The larger of the two numbers is the sample size of this trial. The primary outcome measure is the change in the cumulative duration of oxygen therapy. The cumulative mean duration of severe BPD is reportedly 90 ± 15 days [4, 31]. This trial is powered to identify differences of 15% (from 90 days in the control group to 77 days in the low-dose groups) and 20% (from 90 days in the control group to 72 days in the high-dose groups) in participants who accept the hUC-MSC intervention. Meanwhile, the power is set at 0.8, type I error *ɑ* is 0.05, and type-II error *β* is 0.20. The calculated sample size is 21 for each group. To account for the possibility of 10% of patients being lost to follow-up, the final sample size will be 24 and the total size will be 72.

## Participants

### Patient and public involvement

The participants will not be involved in the development of the trial including the study design, recruitment, and conduct, selection of research question, and outcome measures. The participation will be voluntary and the participants will have freedom to participate or withdraw from this trial at any time throughout the study. The participants’ privacy will be protected.

Participants will be enrolled in this study according to the inclusion and exclusion criteria (Table 1). The parents or guardians of all participants will provide the written informed consent form approved by the Ethics Committee of Stem Cell Clinical Research of CHCMU.

**Table 1:** Inclusion and exclusion criteria for participants with severe bronchopulmonary dysplasia (BPD)

Inclusion criteria	
1. Participants who are male or female and whose age is 0–1 years old 2. Participants who are diagnosed as severe BPD according to diagnostic criteria of BPD made by the National Institute of Child Health and Human Development (NICHD )[5]. 3. Participants who have abnormal respiratory manifestations and the Silverman-Anderson score [32] is more than 3 points 4. Written informed consent signed by a legal representative or a parent	
Exclusion criteria	
1. Participants whose age is more than 1 year old 2. Participants who have no signs of dyspnea or BPD-related changes in pulmonary imaging, such as central apnea or diaphragmatic paralysis although mechanical ventilation or oxygen are required 3. Participants who have concurrent cyanotic or acyanotic congenital heart diseases, except for patent ductus arteriosus, and atrial septal defect and ventricular septal defect with defect < 5 mm 4. Participants whose important laboratory test (liver and kidney functions tests, cardiac markers, hematology and immunity tests, urinalysis, etc.) abnormalities are more than three times compared with the normal value 5. Participants who have severe pulmonary hypertension confirmed by cardiac ultrasound at the time of assessment 6. Participants who have severe respiratory tract malformation, such as Pierre-Robin syndrome, tracheobronchomalacia, vascular ring syndrome, congenital tracheal stenosis, tracheo-esophageal fistula, pulmonary emphysema, pulmonary sequestration, congenital pulmonary dysplasia, congenital pulmonary cyst, congenital spasm, etc. 7. Participants who have severe chromosome anomalies (such as Edward syndrome, Patau syndrome, Down syndrome) or severe congenital malformation (such as hydrocephalus, encephalocele) or hereditary diseases 8. Participants who have severe congenital infection such as *Herpes simplex*, toxoplasmosis, rubella, syphilis, AIDS, etc. 9. Participants who have severe active infection when C-reactive protein (CRP) > 30 mg/dL, or suffer sepsis or septic shock 10. Participants who are going to have surgery within 72 h before/after this study hUC-MSCs administration 11. Participants who have surfactant administration within 24 h before this hUC-MSCs administration. 12. Participants who have severe intracranial hemorrhage ≥ grade 3 or active pneumorrhagia or active air-leak syndrome 13. Participants who are using hormones or needing hormones within and after 7 days of hUC-MSCs administration 14. Participants who are participating in other interventional clinical trials 15. Participants who are considered inappropriate by the investigators or whose parents cannot provide informed consent	

### Inclusion and exclusion criteria

The inclusion and exclusion criteria are listed in Table 1. Participants will be 0–1 year of age. The diagnostic criteria and BPD severity gradation refers to the criteria established by the NICHD workshop [5]. The Silverman and Andersen score is used to assess the severity of abnormal respiratory manifestations [32].

### Recruitment

Patients can only be enrolled in this study after passing the citywide consultation resolution and signing the informed consent form.

Participants will be recruited from three sources. First, the parents of potentially eligible hospitalized patients diagnosed with severe BPD will be approached and asked to join this study. Second, physicians will generate lists of patients from the electronic medical records of CHCMU with a diagnosis of BPD who were discharged within 1 year. Investigators or physicians will contact the patients’ parents by telephone or mail them a research leaflet and recruitment letter. Third, physicians will post study flyers at the outpatient department, the official website, and the WeChat public platform of CHCMU for those diagnosed with severe BPD at other hospitals. If the parents of these patients with severe BPD are interested in this research, we will initiate the screening process.

A multidisciplinary consultation will be held to confirm whether these potential participants meet the general diagnostic criteria of BPD as well as the inclusion and exclusion criteria. The consultation will consist of a neonatologist, a respiratory physician, a radiologist, a laboratory physician, a Department of Critical Care Medicine expert, stem-cell treatment center researchers, and medical department staff. If more than 80% of experts agree on the hUC-MSCs treatment, these patients will be viewed as potential participants. The researcher will then arrange a meeting to communicate with the legal representative or parents about the clinical trial research details and sign the written informed consent form.

The following details of the clinical trial will be fully explained to the patients’ guardians as follows: (1) study purpose; (2) research background; (3) number of participants and duration of their participation; (4) study procedures; (5) potential discomfort and risks of treatment; (6) expected benefits; (7) protection of confidentiality and privacy; and (8) their participation is voluntary. Each patient’s legal representative or parents will sign the informed consent form after all the items above are fully understood. After that, the patients’ baseline characteristics will be recorded by the clinicians (Table 2).

**Table 2:** Timeline and items of evaluation during the trial

Items		Study period									
		Screening phase	Treatment phase				Follow-up phase				
		− 2 weeks	Baseline	24 hours	3 days	7 days	1 month	3 months	6 months	12 months	24 months
Therapy	hUC-MSCs	√	√								
	Traditional treatment	√	√	√	√	√					
Informed consent		√									
Inclusion and exclusion criteria		√	√								
Demographic information		√									
Personal history/past history/family history		√					√	√	√	√	√
Height/weight/head circumference		√	√			√	√	√	√	√	√
Vital signs^a^/physical examination		√	√	√	√	√			√	√	√
Hematology^b^/blood biochemistry^c^/urinalysis^e^		√	√	√							
Infectious disease-related examination^d^		√	√	√							
Blood oxygen saturation/blood gas analysis			√	√	√	√			√	√	√
Chromosome examination		√									
Brain MRI examination		√							√	√	√
EKG		√	√	√							
Echocardiogram		√									
Ventilator parameters/oxygen therapy			√	√	√	√	√	√	√	√	√
Adverse events evaluation			√	√	√	√	√	√	√	√	√
Chest high resolution CT			√						√	√	√
Pulmonary function test			√						√	√	√
Mortality/complications of prematurity^f^							√	√	√	√	√

Legend: *CK-MB* creatine kinase-MB*, CT*, computed tomography *EKG* electrocardiogram, *hUS-MSCs* human umbilical-cord mesenchymal stem cells

^a^The indicators of vital signs include temperature, blood pressure, heart rate, respiratory rate, transcutaneous oxygen saturation

^b^The condition of hematology of participants will be estimated by hematological tests which contains white blood cell count, platelet count, red blood cell count, hemoglobin, the percentage of lymphocytes, the percentage of neutrophil and C-reactive protein (CRP)

^c^The items of blood biochemistry include liver function, renal function, cardiac markers, the indicators of immunity, and the detailed items of each test are listed as follow. Liver function tests: albumin, bilirubin, alkaline phosphatase, alanine aminotransferase, aspartate aminotransferase, prothrombin time, activated partial thromboplastin time. Renal function tests: creatinine, blood urea nitrogen, creatinine clearance, glomerular filtration rate. Cardiac markers: hypersensitive troponin I, CK-MB mass, myoglobin

^d^Infectious disease-related examination include markers of hepatitis/syphilis/HIV/tuberculosis

^e^The content of urinalysis includes pH, protein, specific gravity, glucose and ketone bodies, white blood cells, occult blood or red blood cells, nitrite, color, and turbidity

^f^Complications of prematurity include growth retardation or retardation, hearing abnormalities, retinopathy, pulmonary hypertension, left ventricular hypertrophy

### Randomization and blinding

Participants will be randomized into three groups in a 1:1:1 ratio after collection of the baseline data. The allocation sequence will be generated and sent to the investigators by a statistician. Participants will not be blinded during the phase-II trial and the patients in the control group will not be given hUC-MSC treatment.

### Intervention

The hUC-MSCs produced by Ever Union Biotechnology Co. Ltd. (EUBIO) are transported to the ward on the infusion day. The hUC-MSCs are suspended in 0.9% normal saline. In addition to inspecting the quality of the hUC-MSC product by EUBIO, the staff of the Stem-cell Center in CHCMU will confirm the viability and quality of the hUC-MSC product before the infusion.

There is currently no effective therapy for BPD patients, who are often given traditional supportive treatments such as nutritional support, fluid restriction, and respiratory support (including ventilator support and oxygen supply) so all participants will be given traditional supportive treatments to ensure their safety. Thus, the intervention groups will be given the traditional supportive treatments and extra low- or high-dose hUC-MSC infusion and the control group will be given only the traditional supportive treatment. Participants will be unable to use glucocorticoids 3 days before or after the hUC-MSC treatment.

A total of 72 patients in the intervention groups will be randomized in a 1:1:1 pattern to receive low-dose hUC-MSCs (*n* = 24; 2.5 million cells/kg) or high-dose hUC-MSCs (*n* = 24; 5 million cells/kg) in combination with traditional supportive treatments or traditional supportive treatments alone (*n* = 24).

### Withdrawal

Discontinuation may occur due to participant death, severe adverse effects (SAEs), other serious disease-limiting participation, or study withdrawal requested by the guardian. If the participant withdraws from the trial, the reason for the withdrawal and all the results of observations will recorded in detail. Meanwhile, a new participant will be enrolled in the trial to replace the withdrawn subject.

### Adverse events

AEs are defined as adverse medical events that occur after the subjects or their guardians provide written informed consent until the end of the study visit. AEs include abnormal laboratory results, symptoms, or diseases. All AEs will be recorded on a Case Report Form (CRF) and the researcher should provide comprehensive clinical reports. Once AEs occur, we will follow the principle of “the first priority of the participants,” take the necessary treatment according to the patients’ specific situation, and decide whether to suspend the clinical research. The main investigator should immediately inform the Scientific Research Office, Medical Service, and Ethics Committee of CHCMU. The SAE report should be submitted in writing within 24 h, while a follow-up SAE report should be submitted to the Human Research Ethics Committee.

An insurance policy will be prepared for all participants, who will be provided with ancillary and post-trial care in the case of injury or death as a result of their participation in the trial.

### Outcome evaluation

The outcome measures and their time frames of this trial are listed in Table 3. The primary endpoint is the cumulative duration of oxygen therapy, i.e., the duration from starting to stopping oxygen treatment.

**Table 3:** Outcome measures

Measures			Time frames
Primary outcomes	Cumulative duration of oxygen therapy		Until the time of stopping oxygen therapy
Secondary outcomes	Adverse events	Number of serious adverse events	Within 24 h post hUC-MSCs infusion
		Acute infusion associated adverse events	From the start of the trials to 1 month post hUC-MSCs infusion
		Late infusion associated adverse events	Within 2 years post hUC-MSCs infusion
	The rate of supplemental oxygen therapy		At 1 month post hUC-MSCs infusion
	Duration of invasive mechanical ventilation		
	Duration of noninvasive mechanical ventilation		
	The first time of stopping oxygen supplement		
	The rate of re-oxygen supplement		
	Pulmonary function changes		At 6, 12, and 24 months post hUC-MSCs infusion
	Chest radiography changes		
	Blood oxygen saturation		At 1, 3, 6, 12, and 24 months post hUC-MSCs infusion
	Mortality		
	Times of hospital readmissions		
	Complications of preterm birth		

The secondary endpoints include the safety and efficacy outcomes. The safety of the study will be assessed by the number of AEs including SAEs, acute infusion-associated AEs (AIA-AEs) and late infusion-associated AEs (LIA-AEs). SAEs include death, any malignant cardiac event (new ventricular tachycardia, ventricular fibrillation, or asystole, cardiac arrest), acute pulmonary embolism, stroke, anaphylactic shock, acute transplant rejection, and any other diseases extending the hospital stay. AIA-AEs include fever, general allergic reaction (rash, edema, erythema, pallor), infection at the injection site, vital sign changes, laboratory test changes (indicators of liver and kidney function, cardiac markers, indicators of hematology and immunity, markers of hepatitis/syphilis/HIV/tuberculosis, and urinalysis). LIA-AEs include tumorigenic events (tumor formation) and teratogenic events.

The efficacy endpoints are as follows: duration of oxygen therapy, duration of invasive mechanical ventilation, duration of noninvasive mechanical ventilation, the first time stopping the supplemental oxygen, rate of re-oxygen supplement, blood oxygen saturation, chest radiography changes, pulmonary function changes, mortality, number of hospital readmissions, and preterm birth complications.

### Follow-up procedures

Follow-up assessments will be performed at 1, 3, 6, 12, and 24 months after the hUC-MSC injection (Table 2). The five follow-up points will be conducted by telephone and outpatient contact. The first two follow-ups will be performed at 1 and 3 months after the hUC-MSC treatment by telephone to ask the parents of the condition of their child(ren). The details of the telephone interview are shown in Table 4. The next three follow-ups will be performed at 6, 12, and 24 months at outpatient visits as shown in Table 5. The key outcome during follow-up will be changes on chest radiographs.

**Table 4:**
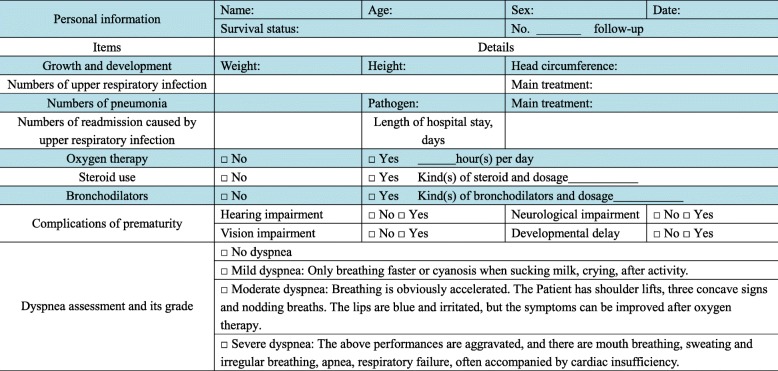
The telephone follow-up list

**Table 5:**
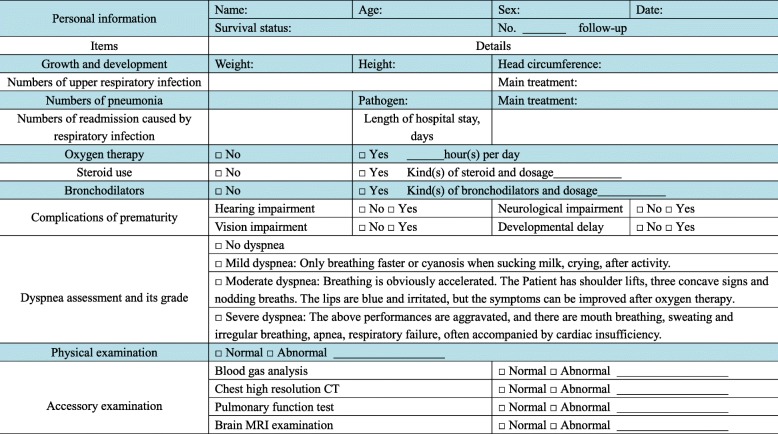
The outpatient follow-up list

Legend; *CT* computed tomography, *MRI* magnetic resonance imaging

### Safety monitoring

The independent Data and Safety Monitoring Board (DSMB) will supervise safety during the trial. The members of DSMB are independent of the sponsor and trial investigators and have no competing interests. The DSMB will review and evaluate clinical safety and efficacy data collected according to the specified time intervals in the protocol. If the threshold of the safety data exceeds a predefined threshold, the DSMB will be notified. Furthermore, the DSMB will conduct the interim analysis of all AE occurrences every 6 months during the study. Only the data managers and study designers have access to the data in the trial. The data will be locked by the Data Management Team when the trial is completed. All of the data will be provided to the DSMB. If the trial is terminated earlier than the expected end date, the DSMB will contribute to that decision.

### Data collection

The data generated during the trial will be recorded in the original medical record and the CRF. The quality control personnel will check the consistency of the CRF data with the original record to ensure that the data are accurately entered into the CRF. There are nine data collection points: baseline, 1 day, 3 days, 7 days, 1 month, 3 months, 6 months, 12 months, and 24 months (Table 2). Within 3 days after completion of the data collection, the research records will be submitted to the research leader for review and all data will be submitted to the project leader within 10 days. Next, the auditor will review each original research record to confirm that the clinical trial data records are timely, precise, and standardized. Data checks and entries will then be disposed by the statistical data manager and analyzed by the statisticians.

The data of this trial will be disseminated through national and international conferences and peer-reviewed publications. Our data set will be available after the trial’s completion.

### Statistical analysis

SPSS version 17 (SPSS Inc., Chicago, IL, USA) statistical analysis software will be used to analyze the data in the study. Significant differences will be considered at an α level of 0.05.

The data will be examined at group assignment to the intervention and control groups using the *χ*^2^ test, *t* test or analysis of variance (ANOVA). For the intervention and control groups, the indicators at baseline and at 1 day, 3 days, 7 days, 1 month, 3 months, 6 months, 12 months, and 24 months will be compared with repeated measures ANOVA. Comparison of these outcome indicators including duration of oxygen therapy, duration of mechanical ventilation, pulmonary function tests, and quantitative scores of chest radiography changes between the intervention and control groups will be conducted using the *t* test. Mortality and hospital readmission rates will be tested by the *χ*^2^ test, while 24-month mortality will be analyzed by Kaplan-Meier curves.

## Discussion

At present, apart from symptomatic supportive therapy, there is no effective treatment for severe BPD patients [33]. Therefore, identifying new treatment methods to improve the prognosis of premature infants is imperative. Studies have shown that stem-cell therapy can significantly improve neonatal hyperoxic lung injury [34, 35], suggesting that stem-cell transplantation may be a promising treatment for severe BPD.

The intratracheal administration of MSCs in premature infants at high risk of BPD has been investigated in several small uncontrolled studies registered at ClinicalTrials.gov. These studies aim to investigate whether local regional hUC-MSC delivery to the airway is safe and potentially effective and could prevent BPD in premature infants. However, most patients with severe BPD only receive oxygen treatment when a medical ventilator was no longer used. Intratracheal hUC-MSC treatment is difficult to administer to patients under these circumstances. Hence, intravenous administration may be a better choice. Recent studies have shown that the intravenous administration of MSCs was safe and had some potential efficacy in several lung diseases, including acute respiratory distress syndrome (ARDS) and chronic pulmonary disease (COPD) [27, 28], but there are few reports on BPD. Based on these studies, the intravenous administration of MSCs is expected to be a worthwhile option for patients with severe BPD. Considering that hUC-MSCs are a main source in clinical trials, this trial aims to evaluate whether allogeneic hUC-MSC therapy is safe and effective in severe BPD patients with a matched control. We hypothesized that this research will provide data showing that hUC-MSC administration is safe and feasible for severe BPD patients.

A variety of studies on hUC-MSC clinical trials performed a dose-escalation of hUC-MSCs of 0.5–5 million cells/kg in adults through intravenous infusions; the largest dose of a few trials reached 10 million cells/kg [27, 28, 36, 37]. However, few trial reports explored the effect of different doses of hUC-MSCs in children’s diseases, especially in those with severe BPD. Thus, we will conduct a dose-escalation trial of intravenously administered hUC-MSCs for the treatment of severe BPD patients. Considering that the features of premature infants include low weight and immature organ function, we determined a maximum hUC-MSC dose of 5 million cells/kg.

Although this planned trial cannot use a high-quality randomized controlled trial design since it is an open-label trial, it has several advantages. First, this is a first trial to investigate the therapeutic rather than preventive effects of hUC-MSCs in children with severe BPD. Second, this study will explore a dose-escalation of hUC-MSC treatment through intravenous administration. Third, if we can observe some efficacy, our results may broaden our understanding of hUC-MSC effects in BPD and provide a basis for treating patients with severe BPD. However, if there are no obvious effects, our study will also have important clinical implications for pediatric refractory diseases. As several kinds of patients currently receive MSC therapy for which potential efficacy has been reported [29], randomized trials are lacking to prove its safety or efficacy.

Overall, the MSC-BPD trial is a vital and exploratory step in the investigation of a new evidence-based therapy for a large number of pediatric BPD patients.

## Trial status

Start date: July 2019.

Expected end date: December 2021.

Status at time of submission of this article: not yet recruiting.

## Supplementary information

**Additional file 1.** Standard Protocol Items: Recommendations for Interventional Trials (SPIRIT) Checklist: recommended items to address in a clinical trial protocol and related documents.

## Data Availability

All the data in the trial will be available for anyone who wants to access the data following publication.

## Abbreviations

AEs: Adverse events
AIA-AEs: Acute infusion-associated adverse events
ANOVA: Analysis of variance
ARDS: Acute respiratory distress syndrome
BPD: Bronchopulmonary dysplasia
CHCMU: Children’s Hospital of Chongqing Medical University
COPD: Chronic obstructive pulmonary disease
DSMB: Data and Safety Monitoring Board
EUBIO: Ever Union Biotechnology Co. Ltd.
hUC-MSCs: Human umbilical-cord mesenchymal stem cells
LIA-AEs: Late infusion-associated adverse events
MSCs: Mesenchymal stem cells
NICHD: National Institute of Child Health and Human Development
SAEs: Severe adverse events
UC: Umbilical cord
